# A personalized real-time virtual model of whole heart electrophysiology

**DOI:** 10.3389/fphys.2022.907190

**Published:** 2022-09-23

**Authors:** Karli Gillette, Matthias A. F. Gsell, Marina Strocchi, Thomas Grandits, Aurel Neic, Martin Manninger, Daniel Scherr, Caroline H. Roney, Anton J. Prassl, Christoph M. Augustin, Edward J. Vigmond, Gernot Plank

**Affiliations:** ^1^ Gottfried Schatz Research Center—Biophysics, Medical University of Graz, Graz, Austria; ^2^ BioTechMed-Graz, Graz, Austria; ^3^ NAWI Graz, Institute of Mathematics and Scientific Computing, University of Graz, Graz, Austria; ^4^ King’s College London, London, United Kingdom; ^5^ NumeriCor GmbH, Graz, Austria; ^6^ Division of Cardiology, Department of Internal Medicine, Medical University of Graz, Graz, Austria; ^7^ Queen Mary University of London, London, United Kingdom; ^8^ Electrophysiology and Heart Modelling Institute, Bordeaux, France

**Keywords:** His-Purkinje system, virtual heart technology, cardiac electrophysiology, 12 lead electrocardiogram, cardiac personalization, cardiovascular disease

## Abstract

Computer models capable of representing the intrinsic personal electrophysiology (EP) of the heart *in silico* are termed virtual heart technologies. When anatomy and EP are tailored to individual patients within the model, such technologies are promising clinical and industrial tools. Regardless of their vast potential, few virtual technologies simulating the entire organ-scale EP of all four-chambers of the heart have been reported and widespread clinical use is limited due to high computational costs and difficulty in validation. We thus report on the development of a novel virtual technology representing the electrophysiology of all four-chambers of the heart aiming to overcome these limitations. In our previous work, a model of ventricular EP embedded in a torso was constructed from clinical magnetic resonance image (MRI) data and personalized according to the measured 12 lead electrocardiogram (ECG) of a single subject under normal sinus rhythm. This model is then expanded upon to include whole heart EP and a detailed representation of the His-Purkinje system (HPS). To test the capacities of the personalized virtual heart technology to replicate standard clinical morphological ECG features under such conditions, bundle branch blocks within both the right and the left ventricles under two different conduction velocity settings are modeled alongside sinus rhythm. To ensure clinical viability, model generation was completely automated and simulations were performed using an efficient real-time cardiac EP simulator. Close correspondence between the measured and simulated 12 lead ECG was observed under normal sinus conditions and all simulated bundle branch blocks manifested relevant clinical morphological features.

## 1 Introduction

Many virtual heart technologies aim to represent the entire organ-scale EP of the heart *in silico* in full mechanistic detail and accordingly provide information of the intrinsic cardiac sources that drive electrical activation and repolarization of the heart. As such, these technologies have the potential to serve not only as surrogates for human clinical and experimental studies, but be a powerful tool within both industrial and clinical applications (Abadi et al., 2020; Corral-Acero et al., 2020). More efficient development of medical devices at cheaper costs, for example, is possible by reducing the number of necessary animal experiments and allowing automated testing of alternative design and deployment variations during the design process. Furthermore, virtual heart technologies could allow optimization of clinical trials and provide patient-tailored therapeutic options (Viceconti et al., 2016). Most importantly, the models can be a transformative tool in clinical diagnostics, prognostics, and treatment planning (Corral-Acero et al., 2020) when personalized. Personalization is performed according to electrical recordings such as the 12 lead ECG and electro-anatomical mappings (EAMs) that are ideally acquired non-invasively.

Despite their potential, current realizations of such technologies fall short in various regards, significantly limiting the scope for potential industrial or clinical applications. Major limitations are the 1) significant computational costs involved in carrying out *in silico* EP studies; 2) the difficulty of personalizing the EP; 3) lack of compelling validation, that is, to demonstrate that simulated EP correlates closely to the physical reality; 4) little proof of predictive capabilities; and, 5) inability to provide estimates of model uncertainties. Furthermore, most reported current technologies focus only on specific chambers of the heart. Namely, studies have reported on either the ventricles (Arevalo et al., 2016; Lopez-Perez et al., 2019) or the atria (Ali et al., 2019; Nagel et al., 2021; Roney et al., 2022), and only a few technologies representing whole heart EP are reported (Strocchi et al., 2020; Gerach et al., 2021; Moss et al., 2021).

We report on the development of our novel virtual technology of whole-heart EP aiming to address these limitations. Our cardiac EP simulator, comprising a whole heart EP model, is able to mechanistically represent the full spectrum of known organ-scale EP under both normal sinus rhythm and bundle branch blocks (BBBs). In previous work, a trimmed down ventricular and torso model of a healthy volunteer was personalized under sinus conditions (Gillette et al., 2021a) and subsequently equipped with a topologically realistic model of the His-Purkinje system (HPS) (Gillette et al., 2021b). Within this study, the model was extended to the whole heart by accounting for atrial EP and adding an atrio-ventricular node (AVN) connected to the HPS facilitating atrio-ventricular conduction. 12 lead ECGs and body surface potential maps (BSPMs) on the torso surface, as well as EAMs recovered through electrocardiographic imaging (ECGi) within the entirety of the heart, were simulated using an efficient and real-time EP simulator first under normal sinus rhythm. The ability of the model to reproduce known ECG features under pathological conditions, which the model was not parameterized for, was then tested. BBBs in the (left and right ventricles LV, RV) were generated. To account for possible remodeling and alterations in the electrical substrate of the ventricles under such conditions (Vernooy et al., 2005; Valenti et al., 2012; Michalski et al., 2022), a lowered (conduction velocity CV) within the associated ventricle was also applied. Close correspondence between simulated and measured 12 lead ECGs is shown under sinus conditions. 12 lead ECGs simulated under pathological conditions under complete BBB are validated based on standard clinical ECG diagnostics (Surawicz et al., 2009).

## 2 Materials and methods

An anatomically-personalized model of a single subject (male, 45 years of age) was built from imaging data and the non-invasive 12 lead ECG, extending on our previous work (Gillette et al., 2021a; Gillette et al., 2021b). Segmentation and meshing was performed semi-automatically to generate a model that explicitly represents all four chambers of the heart, blood volumes, lungs, and torso. Highly automated workflows relying on abstract anatomical reference frames defined within the ventricles and atria separately were then used to define complex space-varying parameters governing cardiac EP over the whole heart. Parameters governing ventricular EP were previously introduced and fitted using a five-fascicular model to represent the HPS (Gillette et al., 2021a). The simplified model was subsequently replaced by an equivalent topologically realistic representation of the HPS to produce a corresponding 12 lead ECG (Gillette et al., 2021b). Atrial EP was prescribed according to experimental and clinical observations. To generate a complete human heartbeat, the proximal HPS was electrically connected to a model of the AVN that was electrically paired to tissue in the basal right atrium (RA). Methodological steps are described in detail below.

### 2.1 Model generation

#### 2.1.1 Anatomical model

An anatomically-specific model of a human whole heart for the single subject was generated from clinical magnetic resonance MRI data (Gillette et al., 2021a). Two separate MRI scans of the full torso and whole heart were sequentially acquired using standardized protocols at 3T (Magnetom Skyra, Siemens Healthcare, Erlangen, Germany). The full torso MRI was attained in four overlapping stacks using a non-ECG gated 3D T1-weighted gradient-echo sequence during expiration at a lower resolution of 1.3 × 1.3 × 3.0 mm^3^. A lower resolution is acceptable as the scan is used purely to construct the volume-conductor model. To preserve anatomical structures of the heart for subsequent segmentation, the whole heart MRI was attained using an ECG-gated, fat-saturated, T2-prepared, isotropic 3D gradient-echo to give a higher output resolution of 0.7 × 0.7 × 0.7 mm^3^. The scan was attained in an expiratory state during free breathing by employing navigators. All subjects gave written and informed consent at the time of the study that was approved by ethical review board at the Medical University of Graz (EKNr: 24–126 ex 11/12).

At the time of image acquisition, a 12 lead ECG was recorded in the supine position using MRI-compatible electrodes. The measured 12 lead ECG had been filtered with a 150 Hz low-pass filter, 0.5 Hz high-pass filter, and a band-stop filter of 50 Hz to remove electrical noise (Kligfield et al., 2007). Electrode positions were later recovered during segmentation.

Four chamber heart segmentation including blood pools from the iso-volumetric cardiac MRI was automatically performed using a two-kernel convolutional neural network originally trained on computed tomography images and boosted for application on MRIs (Payer et al., 2018). The torso MRI was semi-automatically segmented using intensity thresholding in open-source *Seg*3*D* (CIBC, 2016) into heart, lungs, and general torso tissue. Registration using an iterative closest point algorithm was performed to automatically register the four-chamber heart with the heart in the torso segmentation (Chetverikov et al., 2002). Anatomical meshes of all four cardiac chambers were generated from volumetric segmentations using the software *CARPentry-Pro Studio* (NumeriCor GmbH, Graz, Austria). Target resolutions of 1.2 and 4 mm were prescribed for cardiac and torso surfaces, respectively.

#### 2.1.2 Anatomical reference frames

Anatomical reference frames were computed for the ventricles and the atria to facilitate control over spatially-varying EP parameters and to map tissue properties such as fibers. Surfaces and interfaces that were required to define boundary conditions for the computation of the reference frames were automatically extracted from the model using tissue labels and the *meshtool* (Neic et al., 2020) software package. For the ventricles, universal ventricular coordinates (UVCs) were computed within the meshed ventricles according to (Bayer et al., 2018) and modified as discussed in (Gillette et al., 2021a). Universal atrial coordinates (UACs) were assigned to the endocardial surface of the left and right atria as introduced in (Roney et al., 2019; Roney et al., 2021).

#### 2.1.3 Ventricular and atrial fiber architecture

Within the ventricles, fiber architecture was auto-generated using a rule-based method as presented in (Bayer et al., 2012). Ventricular fibers were generated to rotate from +60° on the endocardium to −60° on the epicardium (Streeter et al., 1969). Within the atria, a two-step UAC-based mapping was used to incorporate fiber architecture from an endocardial fiber atlas as in (Roney et al., 2021). In a first step, fibers were mapped from the fiber altas onto the endocardial surface of left and right atria utilizing the UACs generated for our model. In a second step, the fibers were extended transmurally to the volumetric mesh defining the atria. For this reason, a *k*d-tree was generated to map each volumetric cell element in the atrial mesh to the closest surface element on the atrial endocardium. This map was then used to assign the fibers of the surface elements to the related volumetric cell elements. Processing steps of anatomical reference frame generation and fiber integration were also carried out in *CARPentry-Pro Studio* (NumeriCor GmbH, Graz, Austria).

### 2.2 Cardiac electrophysiology

We subsequently detail the EP within the various chambers of the heart, as well as the subsequent simulation of both the cardiac sources and 12 lead ECGs. The pipeline for personalizing and constructing the whole heart model of EP was implemented within the publicly-available *openCARP* simulation framework (Plank et al., 2021). Cardiac simulation was performed in *CARPentry* (Vigmond et al., 2008), which includes implementations and components not available in the *openCARP* simulator, but utilized within this work. Simulation, however, could still be performed using a traditional bidomain simulation approach within the *openCARP* simulator.

#### 2.2.1 His-Purkinje system

A simplified fascicular-based model comprising disk-like root sites embedded in a fast-conducting sub-endocardial layer, representing the fascicles of the HPS in the sub-endocardium, was previously optimized for single subject’s measured 12 lead ECG (Gillette et al., 2021a) and slightly modified within this work. Such a representation modulates the fast spread of activation mediated by the subendocardial Purkinje network, but lacks the ability to capture complex mechanisms during certain disease pathologies and pacing (Gillette et al., 2021b). Therefore, the fitted fascicular-based model was replaced by an auto-generated topologically realistic representation of the HPS. It was previously shown that the 12 lead ECG is preserved during substitution within sinus rhythm (Gillette et al., 2021b).

Improvements were made on the implementation of topologically realistic representation of the HPS to make it compatible for whole heart simulations. Namely, 1) extending the input of the His bundle to include an AVN, 2) alternative branching parameters of 45° repulsion and a mean cable length of 6 mm resulted in a less-dense network allowing faster computation, and 3) incorporating cable-wise tuning of conduction parameters as subsequently described. All fascicles within the HPS were confined to the subendocardium, apart from the fascicle of the RV moderator band, which was allowed to transmurally penetrate up to 50% in the RV free wall to obtain realistic amplitudes in the precordial leads. After discretization to 500 μm, the final HPS contained 958 Purkinje-myocardial junctions and a total of ≈32k nodes. Antegrade and retrograde delays of the HPS were respectively prescribed at 8 and 3 ms (Evans et al., 1984).

CV within the fast-conducting HPS was assumed to be 2.0 m s^−1^ (Kassebaum and Van Dyke, 1966). To ensure activation timings comparable with those directly prescribed in the fascicular-based model, CVs were tuned within the last cable of the HPS preceding the root site of each fascicular branch. In more detail, an initial Eikonal activation map of the HPS was pre-computed to determine the activation timings in the root sites without CV tuning. CVs within each cable preceding a root site were then computed as the Euclidean distance over desired activation delay as prescribed. General myocardial CVs along the longitudinal myocardial fiber orientations were assigned 0.6 m s^−1^ with an off-axis ratio of 4:2:1 (Taggart et al., 2000). Conductivity within the ventricles was set according to (Roberts and Scher, 1982).

#### 2.2.2 Atrial electrophysiology

During sinus rhythm, activation was assumed to start from the sino-atrial node (SAN) placed high on the posteriorly-located crista terminalis (CT) on the roof of the RA after an initial electrical baseline of 25 ms. Within the atrial myocardium, CVs of 1.2 m s^−1^ along the fiber axes with an off-axis value of 0.6 m s^−1^ was assigned in both the RA and the LA. Conductivity values were set using ratios reported in (Roberts and Scher, 1982), but scaled to account for the higher atrial CVs (Roberts and Scher, 1982). To facilitate coordinated activation of the LA and RA, a Bachmann’s bundle (BB) was incorporated into the model that linked the RA from the SAN to the roof of the LA. Between the LA and the RA, the BB wraps between the superior vena cava and the left ventricular outflow tract (LVOT). A higher CV of 2.25 m s^−1^ with an off-axis value of 0.8 m s^−1^ assigned within this region (Nagel et al., 2021). A pathway on the rim of the foramen ovale (Fossa Ovalis (FO)-RIM) was also included on the atrial septum. Additional structures of the SAN block (SAN-B), the pectinate muscles (PMs), and the CT were included, but assigned default EP in accordance with the atrial myocardium.

#### 2.2.3 Cellular electrophysiology and ventricular repolarization

With the aim of keeping simulation times compatible with real-time scales, cellular EP dictating action potential morphology was defined using the Mitchell-Schaeffer phenomenological model within the entirety of the heart (Mitchell and Schaeffer, 2003). This is motivated by the fact that ECG morphology is governed by the spatio-temporal distribution of cardiac electrical sources, which is essentially determined by the CV of travelling depolarization wavefronts and the intrinsic duration of the action potential at a given location in the heart. The Mitchell-Schaeffer model was tuned to the more physiologically detailed Ten-Tusscher model (ten Tusscher et al., 2004; Gillette et al., 2021a). Resulting base parameter values of *ν*_gate_, *τ*_close_, *τ*_in_, and *τ*_out_ were respectively 0.13, 175.0, 0.3, and 5.4. A prescribed resting membrane potential of −86.2 mV and a peak potential of 40 mV were applied.

To establish a physiological T-wave, gradients in the activation recovery intervals (ARIs) across the ventricles were computed assuming a linear mapping with the Eikonal activation map AT(**x**) dictated by ventricular EP,

$$ARI\left(x\right)=m\cdot AT\left(x\right)+b.$$ (1)

The Eikonal activation sequence of only the ventricles was therefore precomputed using the tuned HPS. To compute ARI, a slope of *m* = −0.66 was then used according to (Opthof et al., 2009) and intersect value of *b* = 215 ms was chosen based on the ST interval manually extracted from the measured 12 lead ECG of the subject.

The ARI must then be prescribed using variations in ionic model parameters. The *τ*_close_ parameter of the Mitchell-Schaeffer ionic model primarily dictates maximal action potential duration (APD_max_), a surrogate for the ARI. A relationship between *τ*_close_ and APD_max_ is derived from Eq. (13) in (Mitchell and Schaeffer, 2003), such that

$$\tau_{\text{close}}\left(x\right)=\frac{{APD}_{\text{max}}\left(x\right)}{\ln\left(4\frac{\tau_{\text{in}}}{\tau_{\text{out}}}\right)}\approx \frac{ARI\left(x\right)}{\ln\left(4\frac{\tau_{\text{in}}}{\tau_{\text{out}}}\right)}.$$ (2)

#### 2.2.4 Atrio-ventricular conduction

Atria and ventricles were separated at the base by nodal splitting (Costa et al., 2014) to enforce electrical isolation of the intracellular space. Atrio-ventricular conduction was then mediated by a ventricular conduction system comprising an AVN and a HPS. A topologically simple 1D model of an AVN was incorporated and electrically connected to tissue in the basal RA. CV within the AVN was determined to obtain a physiological PR interval of 200 ms matching the subject’s 12 lead ECG. The CV within the AVN was tuned to a value of 0.07 m s^−1^ to delay the onset of ventricular activation (Efimov et al., 2004). The fast and slow conducting pathways of the AVN were not accounted for. The distal exit of the AVN was connected to the His bundle, mediating propagation into fascicles and Purkinje network. This model of atrial-ventricular conduction facilitates bidirectional electrical conduction across the atrio-ventricular base.

#### 2.2.5 Torso volume conductor model

The lungs, blood pools, and general tissue in the torso volume conductor were set according to 0.0389 S m^−1^, 0.7 S m^−1^, and 0.22 S m^−1^, respectively (Keller et al., 2010). Both the trunk of the aorta and the right ventricular outflow track (RVOT) were integrated into the general torso tissue and electrically inhibited. Electrode placements corresponding to the measured 12 lead ECG were localized to nodes on the torso surface.

### 2.3 Bundle branch block

Disease pathologies of left bundle branch block (LBBB) and right bundle branch block (RBBB) were modeled alongside sinus rhythm under two different CV setups. In both setups, the respective branch of the HPS was inhibited to cause a complete branch block in each case. First, the standard myocardial CV settings were used. As BBBs often cause underlying structural changes or result in cardiac remodelling (i.e. dilation in dyssynchrony) altering activation and conduction within myocardial tissue (Vernooy et al., 2005; Breithardt and Breithardt, 2012; Valenti et al., 2012; Michalski et al., 2022), the longitudinal CV was then reduced to 0.3 m s^−1^ within the affected ventricle while maintaining off-axis ratios. In both conditions, all other EP was assumed to be the same as under sinus rhythm.

### 2.4 Cardiac simulation

Cardiac sources and the potentials at every electrode position for the 12 lead ECG were simulated using the reaction-Eikonal method and lead fields implemented within *CARPentry* (Vigmond et al., 2008; Neic et al., 2017; Potse, 2018; Gillette et al., 2021a). The right leg (RL) electrode was prescribed as an electrical ground for computation. Lead fields were precomputed once for the model and utilized in every pathological simulation. Simulations were conducted for a single heart beat assumed to last 700 ms, in agreement with the subject’s heart rate. A desktop machine with 8 cores was utilized. All simulated ECGs were filtered with a 150 Hz low-pass filter and a high-pass filter of 0.5 Hz to correspond to the measured 12 lead ECG (Kligfield et al., 2007). Note that the 12 lead ECGs are represented in the rotational-axis view.

The reaction-Eikonal simulations were also run in pseudo-bidomain mode to compute extracellular potential fields throughout the heart and torso needed for EAMs. The pseudo-bidomain simulation method, described in great detail elsewhere (Bishop and Plank, 2011; Neic et al., 2017), couples an augmented monodomain model with the elliptic portion of the bidomain model to achieve faster computation of potentials within a bounded simulation domain such as the torso. Speedup is achieved as the elliptic portion is only solved at infrequent time instances dictated by the desired temporal frequency of the electrical signal.

### 2.5 ECG analysis

The 12 lead ECG during sinus rhythm was compared against the measured signal of the subject. For this purpose, the measured signal of 10 s was split into the individual beats by identifying the QRS peaks on lead I and aligning them using a simple thresholding value of 0.2 mV. This allowed us to compute the statistical mean beat against which we compared the simulated ECG. The loss metrics of average L2-norm and the average Pearson correlation coefficient (correlation coefficient (CC)) across all leads in the 12 lead ECG were computed between simulated and mean signals to give an indication of the overall agreement in the signals achieved from the personalization. Scaling of 0.32 is performed to align the signals in terms of amplitude (Gillette et al., 2021a). As no recordings of LBBB and RBBB were available for the healthy subject, QRS duration was computed and the signals were analyzed for standard clinical recommendations set by the American Heart Association for complete LBBB and complete RBBB as reported in (Surawicz et al., 2009). The same scaling is applied under these conditions.

## 3 Results

### 3.1 Workflow performance

**FIGURE 1:**
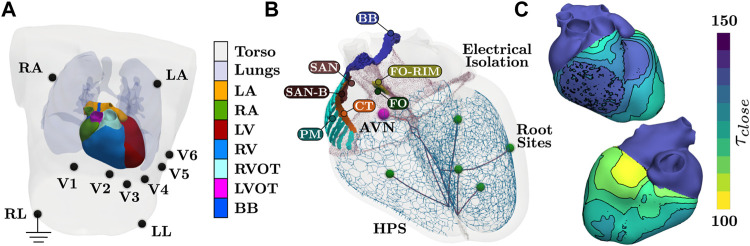
Setup of the torso and whole heart EP simulations. **(A)** Tissue labels present within the model. Blood pools within the heart are not visualized. All electrodes for 12 lead ECG are recovered from the MRI image of the subject, with RL electrode serving as electrical ground. **(B)** Anatomical structures pertaining to cardiac EP. Structures of SAN block (SAN-B), pectinate muscles (PM), and CT are assigned generic EP. The AVN and the SAN facilitate activation, where the BB is assigned a higher CV to facilite coordinate activation of the atria. Each terminal cable before a root site is applied a different CV to control activation timings of the fascicles of the HPS. **(C)** Prescribed *τ*_close_ parameters of the Mitchell-Schaeffer model needed to facilitate a physiological T-wave. Values within the ventricles are computed from the Eikonal activation map. Both atrial tissues and the HPS are assigned the maximal value. Isolines and binning of the color bar indicates 5 ms.

All anatomical entities facilitating cardiac EP could be integrated using abstract reference frames (Figure 1). Due to such high automation in model construction and use of an efficient simulator for cardiac EP, a single human heart beat could therefore be simulated in around 2 min on a desktop machine with 8 cores. Pure model construction time amounted to around 1.5 min, with 9 s denoted to mapping of repolarization heterogeneity and 70 s denoted to construction of the HPS including fascicular CV tuning to keep activation timing optimal at root sites. The remaining model construction time was allocated general setup time. In terms of simulation, computation of all lead fields required a one-time computational cost of around 72 s. Subsequent simulation using the reaction-Eikonal method with the lead fields required approximately 30 s with 10 s purely for mesh IO. Running in pseudo-bidomain mode to recover extracellular potential fields needed for construction of EAMs led to an increased simulation time of 35 min.

### 3.2 Whole heart electrophysiology under sinus rhythm

**FIGURE 2:**
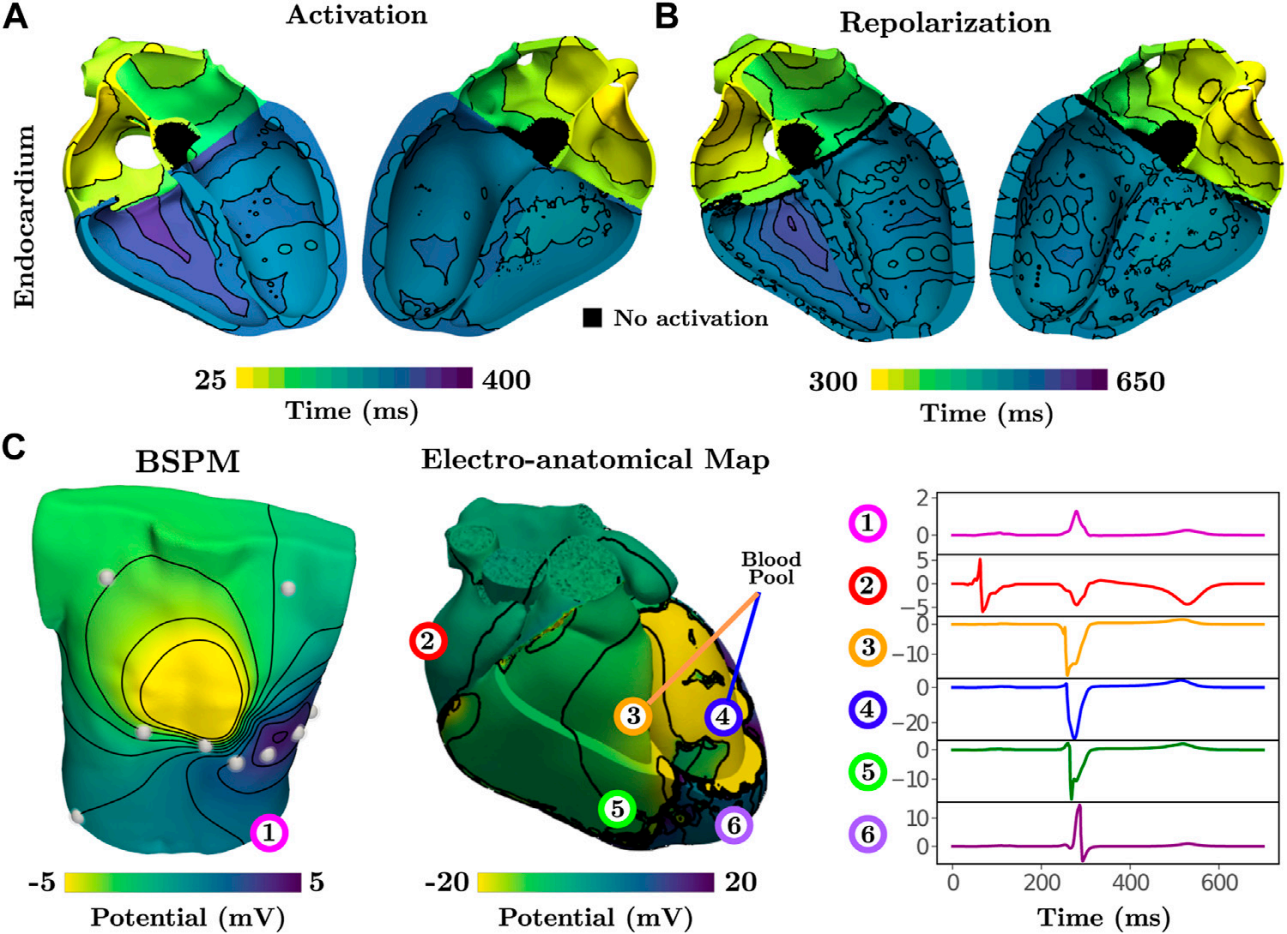
Activation and repolarization patterns during normal sinus rhythm underlying simulated ECGi information of BSPMs and EGMs. **(A)** Activation initiates from fascicular root sites and spreads into the fast-conducting Purkinje network. Latest activation is seen on the posterior RV free wall. **(B)** Repolarization is linearly dependent on activation, thus exhibiting a similar pattern. **(C)** The BSPM and EAM are visualized at a time point of 275 ms. Electrical potentials at 6 recording locations on the torso, on the epicardium, and in the blood pool next to the endocardium are shown for the single heart beat.

Activation of the atria is initiated at the SAN and spreads from the RA to the LA. Inter-atrial conduction within the model is predominately dictated by the BB that leads to earlier conduction on the LA roof. Latest activation is observed in the left-atrial appendage and within the LA just above the base at the LV free wall. Synchronous activation of the LV and RV then ensues due to rapid breakthrough of the fascicles with the myocardium stemming from the root locations. Latest activation occurs on the posterior RV wall due to a lack of Purkinje network within this region (Figure 1B), which is in agreement with reported physiology (Durrer et al., 1970). Due to the dependence of repolarization on activation, similar patterns are observed albeit an inverse relationship (Figure 2B).

Electrical potentials in the form of EGMs reveal the underlying cardiac electrical sources and temporal time sequence when an activation wave-front passes by a given region within the heart. Electrical potentials can also be recovered within the entirety of the model, including on the blood pool and torso surface, to generate information for ECGi, namely BSPMs and EAMs (Figure 2) to replicate all clinically observable EP data.

**FIGURE 3:**
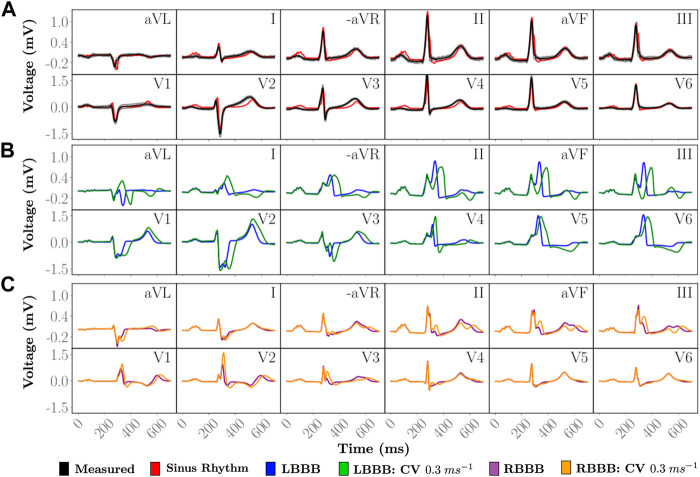
Simulated 12 lead ECGs of a single heartbeat. **(A)** The personalized heartbeat (red) during normal sinus rhythm is compared to an average heart beat in the measured data (black). Gray indicates 2 standard deviations from the average measured signal. Disease pathologies of **(B)** LBBB and **(C)** RBBB are compared to the simulated 12 lead ECG under sinus rhythm. The simulated and measured signals under sinus rhythm have a QRS duration of 85 ms. Simulated LBBB ECGs have QRS durations of 127 and 178 ms with a ...longitudinal CV of 0.6 m/s and 0.3 m/s, respectively, within the LV. Simulated RBBB ECGs have QRS durations of 121 and 148 ms with a longitudinal CV of 0.6 m/s and 0.3 m/s, respectively, within the RV.

Close agreement between simulated and measured ECGs across all 12 leads is attained (Figure 3A). The personalized signal resulted in an average L2-norm difference of 0.08 and a CC of 0.89. Visually, only very minor discrepancies are observed such as the onset of the T-wave in lead V2 or a less pronounced R progression in the precordial leads.

### 3.3 Manifestation of bundle branch block

**FIGURE 4:**
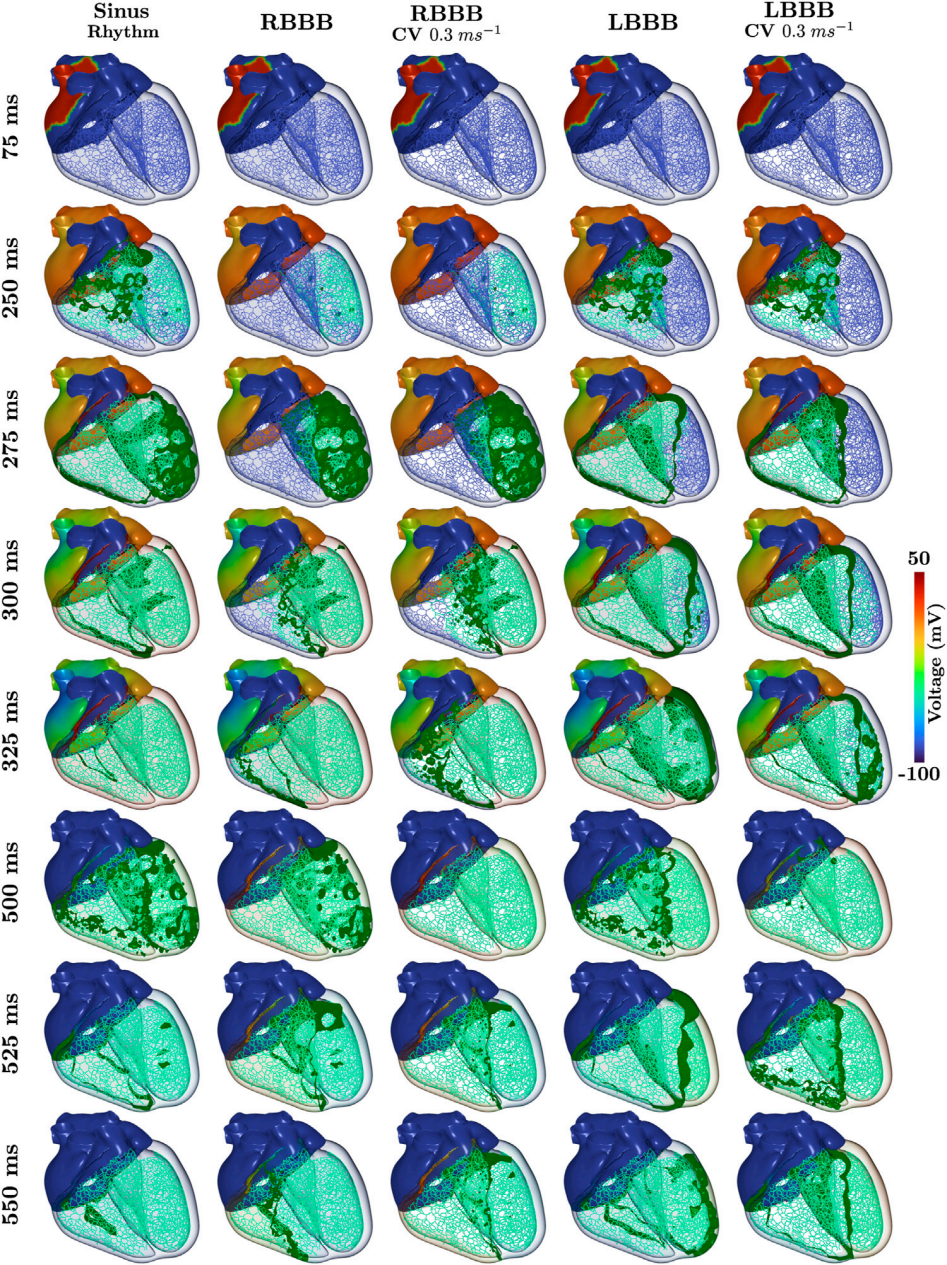
Time-course of the transmembrane voltage within the heart for sinus rhythm and BBBs with both normal and halfed CV in the respective affected ventricle. Green isosurfaces within the ventricles correspond to a membrane voltage of −40 mV and the color map corresponds to the atrial trans-membrane voltages.

Within all cases of BBB, atrial SAN driven activation and associated P-wave remained unaltered. Morphological differences within the 12 lead ECG from normal sinus rhythm (Figure 3) therefore predominantly arise from asynchronous activation of the ventricles as can be observed within the time-course of transmembrane voltages for every pathology (Figure 4). Videos of cellular membrane voltage for both healthy sinus rhythm and BBBs at different CVs are provided that demonstrate the spread of the activation and repolarization wavefronts within the heart. Every video has a temporal rate of 25 frames per second with a total of 700 frames, thus lasting a total of 28.0 s. These videos correspond to Figure 4 within the main text. Corresponding full videos of the membrane voltage through the heart beat are included in the Supplementary Material. While underlying cardiac electrical sources are only shown under sinus rhythm, the same information is available across all pathologies. Standard empirical ECG diagnostics (Surawicz et al., 2009) under complete BBB were used as a metric to gauge the fidelity of the model under disease conditions.

#### 3.3.1 Left bundle branch block

All propagation into the left bundle branch was interrupted to generate a complete LBBB. Activation of the ventricles therefore initiates from the RV. Retrograde activation of the left bundle branch and associated HPS led to a significantly slower activation of the LV as compared to sinus under both CV settings (see 275 ms panels in Figure 4). Apparent morphological features in the 12 lead ECG meet all clinical diagnostic criteria of a LBBB (Figure 3B), and are exaggerated by a reduction in CV within the LV. A prolonged QRS duration of 150 ms ( >120ms ) is attained under LBBB with normal CV that is extended to 195 ms with reduced CV. A deep and broad S with a notch resembling a “W” in leads V1 and V2 and a completely positive and notched R-wave in leads V5 and V6 is also observed. Furthermore, leads I and aVL appear similar to leads V5 and V6. During repolarization, T-wave inversion and ST-segment depression in leads V5, V6, I and aVL in contrast to ST-segment elevation and positive T-waves in leads V1-V3 is also observed.

#### 3.3.2 Right bundle branch block

RBBB was initiated by inhibiting propagation of the right bundle branch of the HPS. Activation within the RV was therefore facilitated by a retrograde activation of the HPS and general myocardial tissues that passes from the LV through the septum (see 300 ms panels in Figure 4). This activation pattern is manifested within the 12 lead ECG (Figure 3C) by a monophasic R in leads V1 and V2, slight slurring of S waves in leads aVL and I, and a QRS duration of 150.0 ms ( >120ms ). With lowered CV within the RV, an inversion in the T-wave in leads V1 and V2 becomes more drastic, as well as an increasing amount of fractionation is observed in leads II, aVL, and III. QRS duration is also extended to 180 ms under reduced CV conditions.

## 4 Discussion

This study reports on the development of a novel torso and whole heart model of EP that is able to compute EGMs and ECGs with real-time performance, thus mitigating typical costs associated with *in silico* EP studies. Anatomically and structurally accurate representations of atria and ventricles were used, which were electrically isolated at the base. A topologically realistic cardiac conduction system was incorporated to electrically connect atria and ventricles, thus mediating bidirectional atrio-ventricular conduction. Personalization of the model was based primarily on the recorded ECG, as well as some experimental and clinical measurements. This novel virtual technology of the whole heart thus constitutes a fully mechanistic model of whole heart EP.

As most current virtual heart technologies reported in the literature do not relate simulated EP to clinically observable data, our novel virtual heart technology constitutes an important advancement towards clinical and industrial *in silico* applications in terms of model fidelity (Viceconti et al., 2016). Mechanistic completeness is demonstrated by modeling two pathologies of the cardiac conduction system, LBBB and RBBB, the model was not parameterized for. Our model is able to replicate intricate morphological features of the 12 lead ECG during both normal sinus rhythm, as well as during LBBB and RBBB according to standard clinical guidelines (Figure 3). The underlying cardiac EP of the given subject must therefore be accounted for to a great extent (Figure 3A). We were able to replicate observable data under sinus rhythm with high fidelity and meet all clinical criteria under BBB, thus building an important foundation for future use of cardiac digital twins (Corral-Acero et al., 2020).

Our methodology of personalizing cardiac digital twins under sinus rhythm and then adapting the model to disease condition could have important implications for predictive and preemptive medicine. The mechanistic completeness endows the model with predictive capabilities, that is, the model can be used under scenarios to which it was not calibrated for, without the need of model re-fitting. Further, model predictions are testable and can be validated by comparing directly to both non-invasive data acquired in clinical routine, or invasive measurements as acquired during an intervention. These capabilities combined facilitate the generation of high fidelity digital twin hearts from a physical patient, open new perspectives in industry, e.g. in the development of device therapies, and in the clinic, for advanced model-based clinical data analysis to support diagnosis, and predictive modeling for planning of optimal therapies.

### 4.1 Workflow performance

Personalization of parameters relating to the QRS complex was previously performed on the simpler BiV model assuming a fasicular-based representation of the His-Purkinje system embedded in a fast-conducting sub-endocardial layer well-suited for efficient simulations at large scale. Extending this representation to the whole heart model, necessary for modeling various disease pathologies, was entirely automatic and required an additional construction time of only 1.5 min. Extension included construction of a detailed His-Purkinje system modulating the same personalized activation pattern under sinus rhythm, and inclusion of the parameters dictating atrial electrophysiology.

In general, lightweight and efficient simulation models are needed to render whole heart simulations tractable and clinically relevant. Simulations of cardiac sources for a single heart beat using the reaction-Eikonal method with lead fields were computationally inexpensive. Recovery of 12 lead ECGs was possible in around 30 s after precomputation of the lead fields requiring approximately 1.5 min. Body surface potential maps and electro-anatomical maps, as well as cardiac sources, needed in various clinical applications, could be computed using reaction-Eikonal in pseudo-bidomain mode in approximately 35 min on a normal desktop machine with 8 cores. Validation of the underlying simulation in previous work reported in Gillette et al., 2021 (Gillette et al., 2021a) showed that ECGs acquired using the reaction-Eikonal method paired with lead fields gave comparable ECG morphologies to a traditional high-resolution, full bidomain simulation at a fraction of the costs. Such fine-detailed full bidomain simulations on even a simpler bi-ventricular setup without a physiologically-detailed His-Purkinje system lasting only 150 ms, required 30 min min on a HPC with 240 computing cores to compute both cardiac sources and potentials used to generate the same 12 lead ECG (Gillette et al., 2021a).

### 4.2 Clinical ECG interpretation

The ECG during sinus rhythm was assessed by making a comparison with the ECG measured from the healthy subject. Close correspondence between the measured and simulated 12 lead ECG could be obtained under normal sinus conditions. As pathological ECGs were not available for healthy volunteer subject, the effect of conduction disturbances, specifically, LBBB and RBBB, was assessed to ascertain the model’s mechanistic completeness. It was demonstrated that all relevant clinical morphological features manifest in the ECG consistent with clinical ECG diagnostic criteria.

#### 4.2.1 Sinus rhythm

The simulated ECGs under sinus rhythm matched the recorded ECG with high fidelity. A very close agreement in ECG morphology across all 12 lead could be attained (Figure 3A) in terms of the averaged L2-norm of 0.08 and CC of 0.89, with all complexes matching in terms of polarity and shape. In the simulated healthy ECG known features manifest such as R-wave progression or the S-wave disappearing between leads V3 to V4. Nonetheless, minor discrepancies are also witnessed that provide hints towards missing features of the model or sub-optimal model parameterization. Overall, QRS morphology is well captured, but R-progression in the precordial leads is slower, with R peaking in V5, not in V4. More striking discrepancies relate to ST-segment and T-wave. Modelled ST-segments differ from clinical ones largely in their slope that is close to zero over prolonged periods. This is less the case with measured ST-segments where a non-zero slope early after QRS initiates a smooth transition to the T-wave. Similarly, the T-wave in the precordial leads features a spikier morphology, with sharper deflections as compared to the ECG.

Discrepancies are anticipated to some extent due to a number of factors. These comprise technological factors related to ECG acquisition such as anatomical uncertainties due to breathing, electrode placement, and signal processing in terms of noise. Modeling factors related to the choice of action potential shape, tissue conductivities, and neglect of structures such as bones, muscle or skin) may also play a role. Finally, the measurement of similarity between ECGs is influenced by the scaling applied (Gillette et al., 2021a). The flat slope of the ST-segment is likely to be related to the Mitchell-Schaeffer model used to model the action potential that lacks features such as early repolarization or a spike-and-dome morphology. In absence of these features, during the plateau phase there is no change in transmembrane voltage, thus minimizing the dynamics of any gradients that could contribute towards an ST-segment slope.

#### 4.2.2 Left bundle branch block

Interrupting the left bundle branch innervating all fascicles in the LV altered the ECG, leading to a morphology that was consistent with all empirically-based standard clinical diagnostic criteria. With LBBB alone, most diagnostic hallmark features could be identified in the simulated ECG, albeit some were borderline. For instance, QRS was prolonged to ≈120 ms, but not beyond to >120ms , as anticipated from a full block of the entire left bundle branch. However, diagnostic criteria are built upon empirical observations from patients suffering from LBBB over prolonged periods of time, not from perfectly healthy subjects with a sudden acutely occurring LBBB, as simulated in this model. Such a persistent ventricular electrical dyssynchrony, associated with changes in electrical activation and repolarization patterns that impairs mechanical performance, triggers downstream remodelling processes (Vernooy et al., 2005; Valenti et al., 2012; Michalski et al., 2022) which may lead to alterations in anatomical shape, size and morphology, in structural changes related to fiber and sheet arrangement and fibrosis, and functional changes in EP affecting action potential shape and duration. These factors combined contribute to clinical LBBB ECGs, but have not been considered.

#### 4.2.3 Right bundle branch block

Similarly to LBBB, interrupting the right bundle branch led to an ECG morphology that was mostly consistent with the ECG criteria for diagnosing RBBB, albeit to a lesser degree than LBBB. In the RBBB case not all diagnostic features manifested after acutely blocking the right bundle branch. For instance, the criterion of a QRS complex in leads V1 and V2 resembling the letter M displaying a rsr’, rsR’ or rSR’ pattern, with rSR‘ being the most common, is not readily apparent. S often does not reach baseline in V1 and V2 which is not the case either. A broad S-wave, with S being longer than R, is only observed in aVL and I, but not in V5 and V6, and S is >40ms in I, but not in V6. Downsloping ST-segments and inverted T-waves are present in leads V1 and V2, but the shape of the T-wave is biphasic, also featuring a positive component. The leads V5, V6, aVL and I all show positive T-waves, consistent with the diagnostic criterion. These discrepancies are explained, analog to the LBBB case, by additional factors beyond the interruption of conduction in the right bundle branch that contribute to the clinical RBBB ECG, but remain unaccounted for in the model.

### 4.3 Validation

Validation of the cardiac sources under all conditions is required. Comparison between the simulated and measured 12 lead ECG signal was only made against the statistical mean beat recorded over 10 s which slightly differs as indicated in Figure 3A. As the inverse problem of cardiac EP is ill-posed, different activation sequences might produce the same ECG. That is, the observation that the computed ECG under a sinus activation replicates the measured ECG of the subject with remarkable fidelity suggests a close relationship in EP between virtual and physical heart, but does not imply that the EP must be the same.

Furthermore, predictive capabilities were only illustrated by assessing the effect of a complete bundle branch block in the virtual heart of a healthy subject according to standard diagnostic criteria as such data cannot be acquired from healthy volunteers. Simulating a wider range of activation sequences under pathologies such as bundle branch blocks that activate the HPS differently, with a fusion of orthodromic and antidromic conduction, is thus required to better interrogate fascicular and network structure of the HPS. While BBB was explored under two different conduction velocity settings, accounting for ventricular hypertrophy, dilation, and remodeling must be explored to better understand the capabilities of the model, as seen in (Galeotti et al., 2013). Simulation of either Wolff-Parkinson-White syndrome or premature ventricular contractions leading to retrograde activation, for example, could also provide valuable insight.

A thorough clinical validation of our model should be performed in patients to better understand the ability to replicate the underlying EP. For example, in patients undergoing His-optimized (HOT) cardiac resynchronization therapy (CRT), a broader range of activation sequences is initiated during implantation, including RV and LV pacing as well as selective or non-selective His and left bundle branch pacing. Such validation is beyond the scope of this study, but digital twin modeling has the potential to revolutionize cardiac resynchronisation therapy by predicting activation patterns, as well as hemodynamics, under different pacing strategies before implantation which would facilitate response-prediction for such costly therapies.

A general limitation of clinical data, however, is related to the inability to observe electrical signals throughout the 3D myocardial muscle. Observations of electrical phenomenon are limited on specific manifolds, such as the body surface or the endocardia where EAMs of relatively high spatio-temporal resolution are recorded during routine clinical mapping procedures. More rigorous experimental validation to determine if the underlying EP is truly representative of the patient could be built on experimental animal studies that acquire electrical data invasively in 3D, throughout the myocardial walls, under a range of induced activation sequences as previously mentioned. Such invasive experimental studies offer a means to record within the entirety of the mammalian heart (Cluitmans et al., 2018; Zenger et al., 2020), and comparison of EGMs could reveal whether the underlying cardiac EP sufficiently approximates the mechanisms within the heart.

Furthermore, as the reported virtual technology of whole heart EP is only personalized for a single subject, however, further validation is required to ensure that the technique for generating a personalized model of cardiac EP is possible in additional subjects. This is particularly important for the generation of model cohorts that could be used for the applications of device development and clinical trials on larger patient groups. Ensuring underlying parameters are capable of representing variation in the patient population is therefore crucial and can be conducted by comparing simulation outputs against databases of 12 lead ECGs such as the publicly available PTB-XL dataset (Wagner et al., 2020). Global sensitivity analysis would also yield valuable information on the relationship between morphological elements in the 12 lead ECG and the input parameter space detailing cardiac EP.

### 4.4 Personalization of cardiac electrophysiology

Simulated physiology correlates closely to the physical reality within the signal subject based on the 12 lead ECG due to model personalization and fitting. Primarily, parameters relating to the location and initiation time of the fascicular sites of the His-Purkinje system had been previously personalized and shown to give improvement within the QRS complex in the 12 lead ECG over an initial fascicular setup (Gillette et al., 2021a). Comparison in activation sequences revealed the underlying differences in the 12 lead ECG were negligible when extending to a more sophisticated representation of the His-Purkinje system used in the study (Gillette et al., 2021b). Various parameters could be tuned using metrics directly taken from the measured 12 lead ECG Namely, the CV of the atrio-ventricular (AV) node, as well as the intersect parameter during ARI mapping for repolarization, could be extracted using the PR and QT intervals, respectively.

Several limitations still exist within this study in terms of modeling personalized cardiac EP, however, as many parameters were assigned based on physiological measurements. Primarily, little personalization of the parameters dictating atrial EP was performed. Locations of the AVN, SAN and atrial structures such as BB were not known within the subject, but were instead generically assigned based on physiological assumptions.

Further, the actual architecture and branching of the HPS is not known and was thus assigned generically. In theory, variation in CV with the branches as detailed in 2.2.1 should indirectly account for differing branching properties that may influence activation breakthrough. Many other components dictating ventricular activation, such as fibers, conductivities, and conduction velocities were also assigned generic values. Regarding the T-wave, the linear slope of the ARI mapping was taken from reported values (Opthof et al., 2009) and the general technique may only be applicable when information on the sinus ECG is available.

## Data Availability

Original contributions presented in the study are summarized in the article. Due to constraints of the IRB approval and subject consent, the ventriculartorso model is not publicly available. The simulation of electrophysiology can be replicated using various tools and simulation code available publicly as part of the open-source *openCARP* simulation framework. Further inquiries about previous work and model availability can be directed to the corresponding author.

## Supplementary material

Supplemental Video S1 Normal Sinus Rhythm.

Supplemental Video S2 Complete LBBB with a normal CV of 0.6 m/s along the longitudinal myocardial fiber orientation assigned in the LV.

Supplemental Video S3 Complete LBBB with a reduced longitudinal CV of 0.3 m/s in the LV.

Supplemental Video S4 Complete RBBB with a normal CV of 0.6 m/s along the longitudinal myocardial fiber orientation assigned in the RV.

Supplemental Video S5 Complete RBBB with a reduced longitudinal CV of 0.3 m/s in the RV.
